# Factors Influencing the Health-Related Quality of Life Among Lower Limb Amputees: A Two-Center Cross-Sectional Study

**DOI:** 10.24248/eahrj.v7i1.718

**Published:** 2023-07-12

**Authors:** Deo J. Hando, Moses J. Byomuganyizi, John B. Ngendahayo, Ramadhani H. Khamisi, Nashivai E. Kivuyo, Peter P. Kunambi, JohnofGod L. Mutajwaha, Giliard R. Mushi, Daniel W. Kitua, Ally H. Mwanga

**Affiliations:** a Department of Surgery, Muhimbili University of Health and Allied Sciences, Dar es Salaam, Tanzania; b Department of Surgery, Muhimbili National Hospital, Dar es Salaam, Tanzania; c Department of Clinical Pharmacology, Muhimbili University of Health and Allied Sciences, Dar es Salaam, Tanzania; d Department of Orthopedics and Traumatology, Muhimbili University of Health and Allied Sciences, Dar es Salaam, Tanzania

## Abstract

**Background:**

Limb amputation is among the commonly performed surgical procedures known to have a significant impact on health-related quality of life (HRQoL). Nonetheless, factors influencing the HRQoL among amputees have not been extensively explored. We therefore conducted a study aiming at determining factors influencing the HRQoL among lower limb amputees.

**Methods:**

A cross-sectional study was conducted from May 2021 to December 2021 in two specialized hospitals located in Dar es Salaam, Tanzania. A total of 160 participants who exclusively underwent lower limb amputation(s) were recruited. Data was collected using a checklist incorporating the 36-Item Short Form Survey (SF-36) questionnaire. Multivariable linear regression analysis was performed to identify factors influencing the HRQoL.

**Results:**

The mean age of the study participants was 53.8 (±15.44) years; with males constituting 68.7%. The mean duration since amputation was 19.84 (±12.44) months. A relatively poor physical component summary score (PCS), as opposed to the mental component summary score (MCS) of the SF-36 was observed among the participants (42.0 vs. 59.3, respectively). Factors that positively influenced the PCS included achieving a college/university level of education, absence of stump pain, and the use of a prosthetic device or crutches. Conversely, factors that negatively influenced the PCS included increasing age and the absence of associated comorbid conditions. These factors accounted for 34.1% of the variance in the PCS. With reference to the MCS, post-amputation employment, longer durations since amputation, and the use of prostheses or crutches had a positive influence. However, having no associated comorbidity negatively influenced the MCS. These factors explained 26.5% of the variances in the MCS.

**Conclusion and Recommendations:**

The aforementioned factors should be addressed accordingly in order to ensure a holistic approach in the management of lower limb amputees. Moreover, longitudinal studies are recommended to systematically study the change in HRQoL over time and to assess its determinants.

## INTRODUCTION

Limb amputation has been a commonly performed surgical procedure since its first description by Hippocrates in 460 to 377 BC.^1^ Despite the therapeutic intent of undertaking this procedure, the morbidity associated with limb loss is shown to have a significant social, economic, and psychological impact not only on the affected individuals but also on the community.^2^

Timely and comprehensive rehabilitation services are pointed out to be pivotal in facilitating adjustment/coping with limb loss, social integration, duty independence, and return to productive life; all of which have a profound influence on the Quality of Life (QoL).^3–6^ Several sociodemographic and amputation-related factors are reported to influence QoL among amputees. Some of these factors include; age, level of education, time since amputation, level of amputation, level of physical activity, associated comorbidities, use of prostheses/assistive devices as well as the duration and severity of stump/phantom pain.^7,8^

The QoL among lower limb amputees in Tanzania and equally so in most developing countries is reported to be poor.^9^ Furthermore, studies examining factors that influence the QoL among amputees in low-income settings are scarce.^10^ With such apprehension, this study was conducted to determine factors that influence Health-Related Quality of Life (HRQoL) among Lower Limb Amputees (LLAs) in Tanzania. The identification of these factors will contribute to the development of comprehensive strategies for managing LLAs, thereby improving their QoL.

## METHODS

### Design and Settings

This was a cross-sectional study conducted at Muhimbili National Hospital and Muhimbili Orthopedic Institute from May 2021 to December 2021. The 2 tertiary referral hospitals located in Dar es Salaam, Tanzania provide specialised care to patients from across the country and from neighboring East and Central African countries.

### Participant Selection and Sample Size

To maximise the precision of the study findings and eliminate sampling variability, the study included all patients aged 18 years and above who underwent lower limb amputation(s) between January 2018 and July 2021. However, patients with concurrent upper extremity amputation, hearing or speech impairments, and mental incapacitation were excluded from the study.

A multiple regression power analysis was performed, utilising the methods described by Cohen (1988).^11^ The analysis aimed to determine the appropriate sample size so as to achieve a high level of statistical power. It was determined that a sample size between 80 and 160 participants would yield a statistical power ranging from 81% to 100%. This level of power would enable the detection of coefficient of determination (R^2^) values ranging from 0.16 to 0.46 which can be attributed to 6 independent variables.^7^ The statistical test employed in this analysis was an F-Test, with a significance level (alpha) set at 0.05.

### Data Collection

Data was collected using a checklist that incorporated the 36-Item Short Form Survey (SF-36) questionnaire (English and Swahili versions). Clinical information was retrieved from the medical records whereas socio-demographic information, symptomatology, and the HRQoL were assessed via over-the-phone interviews.

The SF-36 questionnaire was used to evaluate the post-amputation HRQoL. The questionnaire is categorised into 8 domains (35 items); assessing physical function (10 items), role limitations due to physical health problems (4 items), bodily pain (2 items), general health (5 items), vitality (4 items), social functioning (2 items), role limitations due to emotional problems (3 items) and emotional well-being (5 items). The 8 domains are aggregated into 2 summary measures; the Physical Component Summary (PCS) and Mental Component Summary (MCS) scores.^12,13^

The PCS and MCS were treated independently as the outcome variables. The predictor variables included the socio-demographic characteristics and amputation-related characteristics of the participants.

### Statistical Analysis

Cross-checking of the filled checklists for data completeness was performed for quality control. Data was entered and analysed using IBM SPSS Statistics, Version 26.0. Armonk, NY. The descriptive demographic and amputation-related characteristics were presented as frequencies, proportions, and means (standard deviation [SD]). The PCS and MCS scores derived from the 8 scales of SF-36 were obtained by re-coding the specific composite ‘items’ to create a summative score with a scale ranging from 0 to 100 for both component summary scores (PCS and MCS). Low scores indicated more disability whereas high scores indicated less disability. Forward stepwise multivariable linear regression was performed to assess for factors influencing PCS and MCS. The statistical significance was set at *p*<.05.

### Ethical Consideration

Ethical clearance was obtained from the Institutional Review Board of the Muhimbili University of Health and Allied Sciences (Ref. No.DA.282/298/01.C/). A verbal informed consent was sought from the participants prior to commencing the over-the-phone interviews and the consent process was documented for each participant. The study was performed in accordance with the ethical standards laid by the 1964 Helsinki Declaration and its later amendments on comparable ethical standards.

## RESULTS

### Socio-Demographic Characteristics

**FIGURE 1:**
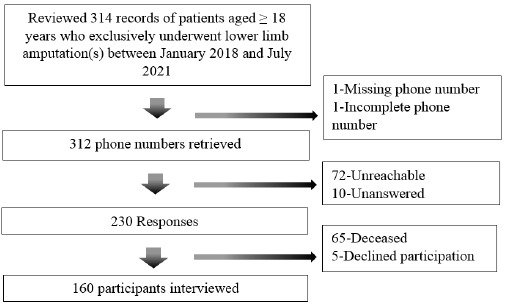
Recruitment Flowchart

The socio-demographic characteristics of the study participants are presented in Table 1. The flow of selection of 160 LLAs included in the study is depicted in Figure 1. Of the 160 participants, males (68.7%) accounted for more than two-thirds of the participants. The mean age of the sample population was 53.8 (±15.4) years. Majority of the participants were married (81.2%) and resided in urban areas (76.9%). Over 90% of the participants had formal education. A relative decline (46.8%) in the proportion of employed participants was observed in the post-amputation period.

**TABLE 1:** Socio-Demographic Characteristics (n=160)

Variables	Frequency (%) / Mean (±SD)
Age (years)	53.8 (± 15.4)
Sex	
Male	110 (68.7%)
Female	50 (31.3%)
Marital status	
Single	16 (10.0%)
Married	130 (81.2%)
Divorced	8 (5.0%)
Widow or Widower	6 (3.8%)
Residence	
Urban	123 (76.9%)
Rural	37 (23.1%)
Level of education	
No formal education	15 (9.4%)
Primary education	81 (50.6%)
Secondary education	51 (31.9%)
College/University	13 (8.1%)
Pre-amputation employment status	
Unemployed	39 (24.4%)
Employed	121 (75.6%)
Post-amputation employment status	
Unemployed	114 (71.2%)
Employed	46 (28.8%)

Key: SD, Standard deviation

### Amputation-Related Characteristics

The amputation-related characteristics of the study participants are presented in Table 2. The mean duration since amputation was 19.84 (± 12.44) months. Diabetes Mellitus was the most commonly reported comorbid condition, with diabetic foot ulcers accounting for about two-thirds of the indications of amputation. Over 95% of the amputations were unilateral, with trans-tibial (46.3%) and trans-femoral (47.5%) accounting for the majority. Ninety-one percent of the participants underwent postoperative physiotherapy. Stump skin complications, stump pain, and phantom limb sensation were reported in 10.0%, 43.1%, and 37.5% of the amputees respectively. Only 21.9% of the participants used a prosthetic device; whereas, over 90% used assistive devices.

**TABLE 2:** Amputation-Related Characteristics (n=160)

Variables	Frequency (%) / Mean (±SD)
Duration since amputation (months)	19.84 (± 12.44)
Associated comorbid conditions	
None	47 (29.3%)
Diabetic Mellitus	62 (38.8%)
Hypertension	14 (8.8%)
Diabetic Mellitus and Hypertension	36 (22.5%)
Chronic kidney disease	1 (0.6%)
Indication for amputation	
Trauma	16 (10.0%)
Diabetic foot ulcer	103 (64.4%)
Non-diabetic foot infections	17 (10.6%)
Wet/dry gangrene secondary to Peripheral vascular disease	13 (8.1%)
Malignancy	11 (6.9%)
Type of amputation	
Unilateral	156 (97.5%)
Bilateral	4 (2.5%)
Level of amputation	
Trans-tibial	74 (46.3%)
Trans-femoral	76 (47.5%)
Knee disarticulation	1 (0.6%)
Syme	7 (4.4%)
Hip disarticulation	1 (0.6%)
Chopart	1 (0.6%)
Post-amputation physiotherapy (Yes)	48 (91.2%)
Stump skin complications (Yes)	16 (10.0%)
Stump pain (Yes)	69 (43.1%)
Phantom limb sensation (Yes)	60 (37.5%)
Use of prosthesis (Yes)	35 (21.9%)
Use of assistive devices	
None	14 (8.8%)
Wheel-chair	21 (13.1%)
Crutches	125 (78.1%)

Key: SD, Standard deviation

### Description of the SF-36 Domains and Component Summaries

Table 3 summarises the mean scores of the SF-36 domains and component scores attained by the study participants. The overall mean PCS was below average (<50). Low domain scores attained in physical functioning (30.34), and role limitations due to physical problems (4.88) contributed to the overall low PCS (42.03). However, above-average scores (>50) attained in all respective domains resulted in a relatively high MCS (59.37).

**TABLE 3:** Stratification of the SF-36 Domains and Component Scores of the Amputees (n=160)

SF-36 domains	Mean (±SD)
Physical functioning^a^	30.34 (21.39)
Role limitations due to physical problems^a^	4.88 (11.01)
Bodily pain^a^	81.23 (18.80)
General health^a^	51.65 (19.18)
Vitality^b^	56.14 (22.32)
Social functioning^b^	54.85 (40.45)
Role limitations due to emotional problems^b^	60.23 (47.95)
Mental health^b^	66.27 (15.80)
**Component scores**	
Physical component summary score (PCS)^c^	42.03 (11.68)
Mental component summary score (MCS)^c^	59.37 (23.60)

Key: SD, Standard deviation; n, sample size

a Domains contributing to the PCS;

b Domains contributing to MCS;

c Scores range from 0-100

### Factors Influencing Health-Related Quality of Life

The final linear regression model of the factors influencing HRQoL among the LLAs is presented in Table 4. Several factors were found to have a positive impact on the PCS of HRQoL. These factors included; achieving a higher education level, absence of stump pain, and utilisation of a prosthetic device or crutches. On the other hand, increasing age and absence of associated comorbid conditions were identified as factors that negatively influenced PCS. Collectively, these predictors explained 34.1% of the variance in PCS.

**TABLE 4:** Multivariate Linear Regression Model of Factors Influencing the Health-Related Quality of Life (n=160)

Outcome variables	Predictors	β	SE	R^2^	p-value
SF-36 PCS	Model Summary		9.663	0.341	<.001
	Constant	39.683	4.257		
	Age	−0.133	0.057	0.023	.022
	College/University education level	7.237	2.922	0.032	.014
	No comorbidity	−5.515	1.856	0.021	.003
	No stump pain	7.680	1.568	0.126	<.001
	Use of prosthesis	7.098	1.902	0.083	<.001
	Use of crutches	5.194	1.946	0.056	.003
SF-36 MCS	Model Summary		20.563	0.265	<.001
	Constant	40.397	4.758		
	Employed (post-amputation)	10.545	3.797	0.061	.006
	No comorbidity	−9.752	3.659	0.029	.009
	Duration since amputation	0.500	0.139	0.132	<.001
	Use of prosthesis	8.621	4.021	0.022	.034
	Use of crutches	8.958	4.011	0.021	.027

Key: β, Unstandadised beta coefficient; MCS, Mental component summary score; PCS, Physical component summary score; R^2^, Coefficient of determination; SE, Standard error

Regarding the mental component of the HRQoL, employment during the post-amputation period, longer duration since amputation, and the use of prostheses or crutches had a positive influence on MCS. However, the absence of associated comorbidities had a negative influence on MCS. These predictors explained 26.5% of the variances in MCS.

## DISCUSSION

We presented the findings of 160 patients who underwent lower limb amputation(s) in 2 tertiary referral hospitals located in Dar es Salaam, Tanzania. The mean age (middle-aged adults) and gender distribution (males > females) of the study participants were in keeping with findings from previous studies.^7,9,14^ The performance of the physical (PCS) and mental (MCS) health dimensions of the HRQoL were scored 42.03 and 59.37 out of 100, respectively. When compared to the general population, the PCS scores were relatively low (42.03 vs. 54.7); whereas, the MCS scores remained more or less unaffected (59.37 vs. 55.5).^15^ This indicated that lower limb amputation had a major impact on the physical domain of health as opposed to the mental domain. However, dissimilarities in the studies' methodological approaches might also account for these variations.

Results from this study also provide evidence of factors that influence HRQoL among LLAs. Findings suggest that the status of associated comorbid conditions and the use of prosthetics or assistive devices have a significant influence on both the physical (PCS) and mental (MCS) aspects of health. However, age, level of education, and stump pain only influenced the PCS; whereas the post-amputation employment status, and duration since amputation exclusively influenced the MCS.

Advancing age has been associated with an increased risk of diseases and disabling conditions, both of which are proven to have a negative influence on the QoL.^16,17^ However, when examined independently, advancing age and the absence of comorbid conditions exhibited a synergistic influence on the HRQoL among the LLAs. The absence of comorbid conditions had a negative influence on both aspects (PCS and MCS) of HRQoL; while advancing age had a negative influence, particularly on the physical aspect of health (PCS). The earlier finding could most likely be pronounced among patients who were apparently well and underwent amputation secondary to an acute event i.e. trauma. For the later finding regarding advancing age, a negative impact has also been observed in previous studies.^18–20^ This might be explained by the presence of other comorbid conditions that further impair mobility with subsequent impairment of physical health.

Prostheses and assistive devices play an integral role in post-amputation rehabilitation. The use of prosthetic devices has been reported in various studies to have a positive impact on QoL.^10,18^ Notwithstanding, a significant difference in occupational performance and satisfaction among lower limb amputees has been observed with the use of different types of assistive devices.^21^ In this study, both prostheses and assistive devices, particularly axillary crutches had a positive impact on HRQoL. The maintenance, restoration, and improvement of function aided by these devices could explain the findings. Nonetheless, several amputation-related complications including stump skin complications (10.0%) and residual stump pain (43.1%) reported among the study's participants could have limited the use of prostheses with subsequent impairment of HRQoL. Supporting this argument, the absence of stump pain was found to have a positive influence on the physical health domain among the study participants. Similar findings are also reported in previous studies.^7,22^

The level of education as part of a broad and complex socio-economic domain may significantly influence post-amputation rehabilitation and long-term care of amputees.^23^ High levels of education have been linked to better levels of HRQoL, with the biggest impact observed on the mental health dimension.^24^ However, in this study, a positive impact was observed on the physical health dimension. Findings also revealed that only 8.1% of the study participants had achieved a university/college level of education, suggesting that majority of the rest might have been engaging in physically demanding activities prior to amputation. This might explain the high rate of loss of employment observed among the study participants during the post-amputation period as retaining a physically demanding job following amputation is challenging.^5,7^ An alternative explanation for the fall in the levels of employment could be based on the average time taken to Return to Work (RTW) following amputation. Previous studies have described a 2-year mean duration of the time taken to RTW^5,25,26^, which on average is longer than the ‘duration since amputation’ of the study participants. Nonetheless, independent of the duration taken to RTW, post-amputation employment had a positive influence on the mental aspect of health.

A longer duration since amputation was also observed to positively influence the mental health of the study participants. This can be linked to the RTW concept discussed earlier or explained by the fact that longer post-amputation duration brings about better adjustments to the post-amputation condition.^14,27,28^

## CONCLUSION AND RECOMMENDATIONS

The findings revealed that LLAs had a lower PCS compared to MCS score. This signifies marked limitations in physical activities among LLAs. Our study also showed that having completed university/college education, securing an employment post-amputation, experiencing no stump pain, and longer time since amputation had a positive effect on the HRQoL. Conversely, older age and the absence of comorbidities were associated with a negative impact on the HRQoL.

Enhancing the HRQoL for LLAs requires a comprehensive and holistic approach that involves healthcare providers with expertise in rehabilitation, psychology, social work, and other relevant fields. This multidisciplinary approach should be implemented throughout all phases of care provision and should be integrated into standard care protocols. To be effective, the approach should address physical, psychological, and social factors that impact the HRQoL. Moreover, Longitudinal studies are recommended to systematically study the change in QoL over time and to assess its determinants.

### Study Limitations

Some of the inherent limitations of the study design include failure to assess the pre- vs. post-amputation change in the HRQoL or compare the HRQoL between the participants and the general population. Additionally, the design could not demonstrate the temporal relationship between the dependent and independent variables.
